# Aberrant cortical surface and cognition function in drug-naive first-episode schizophrenia

**DOI:** 10.1186/s12991-022-00381-7

**Published:** 2022-02-10

**Authors:** Qianqian Wei, Wei Yan, Rongrong Zhang, Xuna Yang, Shiping Xie

**Affiliations:** 1 Department of Psychiatry, Affiliated Nanjing Brain Hospital, Nanjing Medical University, Nanjing, 210029 China; 2 Medical Department, Suzhou Guangji Hospital, Suzhou, 215008 China

**Keywords:** Schizophrenia, Drug-naive, First-episode, Cognition, Cortical surface

## Abstract

**Objective:**

Impaired cognitive function is a central symptom of schizophrenia and is often correlated with inferior global functional outcomes. However, the role of some neurobiological factors such as cortical structure alterations in the underlying cognitive damages in schizophrenia remains unclear. The present study attempted to explore the neurobiomarkers of cognitive function in drug-naive, first-episode schizophrenia by using structural magnetic resonance imaging (MRI).

**Methods:**

The present study was conducted in patients with drug-naive, first-episode schizophrenia (SZ) and healthy controls (HCs). MRI T1 images were pre-processed using CAT12. Surface-based morphometry (SBM) was utilised to evaluate structural parameters such as cortical thickness and sulcus depth. The positive and negative syndrome scale (PANSS) and Chinese version of the Measurement and Treatment Research to Improve Cognition in Schizophrenia (MATRICS) consensus cognitive battery (MCCB) were employed to estimate the psychotic symptoms and cognition, respectively.

**Results:**

A total of 117 patients with drug-naive first-episode schizophrenia (SZ) and 98 healthy controls (HCs) were included. Both the cortical thickness and sulcus depth in the frontal lobe were lower in patients with SZ than in the HCs under family-wise error correction (*p* < 0.05). Attention and visual learning in MCCB were positively correlated with the right lateral orbitofrontal cortical thickness in the patients with SZ (*p* < 0.01).

**Conclusions:**

The reduced surface value of multiple cortical structures, particularly the cortical thickness and sulcus depth in the frontal lobe, could be the potential biomarkers for cognitive impairment in SZ.

## Introduction

Schizophrenia, a persistent and disabling psychiatric disorder, affects 0.5–1% of the global population and is commonly characterised by a range of positive, negative, and cognitive symptoms [1, 2]. Although positive symptoms in the form of hallucinations and delusions are the most distinctive features of the disorder, cognitive impairment usually precedes the onset of psychosis and is a reliable predictor of the overall long-term social functional outcome [3]. Cognitive domains such as working memory, executive function, and attention are generally damaged in schizophrenia [4, 5]. Several studies have exhibited that cognitive impairments such as decline in executive function occur in the initial stages or at the onset of schizophrenia [6]. Cognitive impairment is observed throughout the course of schizophrenia, and the degree of cognitive recovery is strongly associated with the individual prognosis [7].

The Measurement and Treatment Research to Improve Cognition in Schizophrenia (MATRICS) Consensus Cognitive Battery (MCCB) is a typical and representative cognitive testing tool that has been extensively employed in China to assess the cognitive function in patients with neuropsychiatric disorders such as schizophrenia and bipolar disorder [8]. The reliability and validity of MCCB have been verified through the development and revision by researchers, system testing, and clinical trials [9]. Numerous studies have demonstrated that patients with both first-episode and chronic schizophrenia exhibit cognitive deficits in multiple cognitive functions evaluated using MCCB [10, 11].

Several animal and cadaver brain studies support the neurodevelopmental hypothesis of schizophrenia, which indicate that the brain development in schizophrenia is abnormal [12, 13]. MRI studies have demonstrated the aberrant structure and function of the brain in schizophrenia, including structural abnormalities such as altered grey matter volume [14], thickness, and gyrification [15]. Some variations in the grey matter volume or thickness of the brain are significantly associated with cognitive damage in patients with schizophrenia, and the abnormal frontal and temporal lobes are commonly considered the critical areas [16]. The results of studies that have explored the neurodevelopmental mechanisms of schizophrenia have been inconsistent. Heterogeneity among these studies may be attributed to the factors such as variations in medications, multiple-episode courses, duration of illness, and study samples.

Thus, the present study was conducted in patients with drug-naive first-episode schizophrenia (SZ) and healthy controls (HCs). MCCB was used to assess cognition, and surface-based morphometry (SBM) was used to investigate the structure of cortical matter in patients with SZ and HCs. The grey matter microstructure can be quantitatively assessed through voxel-based morphometry (VBM) and SBM. SBM is more sensitive and better at detecting the brain structure variation than VBM. The present study performed a hypothesis-free analysis and attempted to explore the altered brain grey matter based on cortical thickness and sulcus depth in patients with SZ and HCs. Furthermore, the study attempted to identify the specific brain grey matter aberrations that are responsible for causing cognitive deficits in patients with SZ.

## Materials and methods

### Subjects

The present study was conducted in drug-naive first-episode schizophrenia (SZ) and healthy controls (HCs). SZ in all the patients was diagnosed by two experienced psychiatrists according to the criteria specified in the diagnostic and statistical manual of mental disorders (DSM)-IV. Inpatients or outpatients in Nanjing Brain Hospital, Jiangsu, China, were included in the study. All the patients meeting the eligibility criteria were voluntarily recruited continuously from 2016 to 2020. Right-handed patients between 16 and 45 years of age, with an intelligence quotient (IQ) of > 70 and a duration of untreated SZ ≤ 24 months, who were never treated with antipsychotic medications or physical therapy were included in the study. Pregnant women, patients with a history of mental illness, and those contraindicated for magnetic resonance imaging (MRI) scanning were excluded from the study. The clinical trial was approved by the Institutional Review Committee of the Affiliated Brain Hospital of Nanjing Medical University. All experimental procedures were performed in accordance with the Declaration of Helsinki and other relevant regulations. Written informed consent was obtained from all the participants before conducting the study.

All HCs in the present study were enrolled consecutively from the community through poster advertisements. The absence of psychosis in the HCs was confirmed by a professional psychiatrist who used the Structured Clinical Interview for DSM-IV-TR Axis I disorders: Patient edition. Additionally, the HCs were examined for the absence of any significant medical or neurological illness, pregnancy, suicidal tendency, history of alcohol or drug abuse, or MRI scanning contraindications.

### Clinical measurement

Clinical demographic information was provided by the patients and their caregivers. The right-handedness of all patients was determined using the Oldfield Handedness Questionnaire. The IQ and psychopathology outcomes of the participants were estimated using the Wechsler adult scale of intelligence and the positive and negative syndrome scale (PANSS) comprising three subscales, namely positive symptoms (PANSS-P), negative symptoms (PANSS-N), and general psychiatric symptoms (PANSS-G) [17]. Repeated assessment for the PANSS total score maintained an inter-rater correlation coefficient of > 0.8. The Chinese version of the MCCB includes a measure of cognitive function, and the final scores were adjusted for age, sex, and years of education. MCCB comprises the following seven psychological dimensions and 10 sub-tests [18]: (1) processing speed: trail making test, digit symbol coding subtest, and semantic verbal fluency test; (2) attention/vigilance: continuous performance test-identical pairs; (3) working memory: digital sequencing test and space span test; (4) verbal learning and memory: Hopkins verbal learning test-revised; (5) visual learning and memory: brief visuospatial memory test-revised; (6) reasoning and problem solving: mazes test; (7) social cognition: emotion management test.

### MRI data pre-processing

All MRI data were obtained from the Nanjing Brain Hospital, Jiangsu, China, and an MRI system at 3.0  T (Siemens, Skyra, Germany) was applied. The following MRI parameters of the 3D T1 gradient echo were recalled in (fast field echo) sequence: repetition time (TR) = 2500 ms, echo time (TE) = 2.96 ms, flip angle (FA) = 9°, field of view (FOV) = 256 × 256 mm, slice per slab = 192, voxel size = 1.0 × 1.0 × 1.0 mm, slice thickness = 1.0 mm.

### Data analysis

The independent sample *t*-test, analysis of variance (ANOVA), and the Chi-squared test were used to compare the demographic and clinical data. SPSS version 25.0 was used for data analyses.

The Computational Anatomy Toolbox for SPM (CAT12) was used to process cortical thickness and sulcus depth [19]. The specific process is as follows: 1. pre-processing: cortical surface estimation, topological correction, and spherical mapping or registration; 2. index extraction: cortical thickness and sulcus depth were resampled or smooth surfaces by using 15- and 20-mm full width at half maximum (FWHM) Gaussian kernel, respectively; 3. statistical analysis: analysis of covariance (ANCOVA) was used to compare differences between patients with SZs and HCs by utilising age, education level, and sex as covariates, and family-wised error (FWE) correction was used for multiple comparisons (*p* < 0.05) [20, 21]. Additionally, the Pearson correlation test was performed for assessing the association among values of different brain regions and clinical data.

## Results

### Demographic and clinical measures

**Fig. 1:**
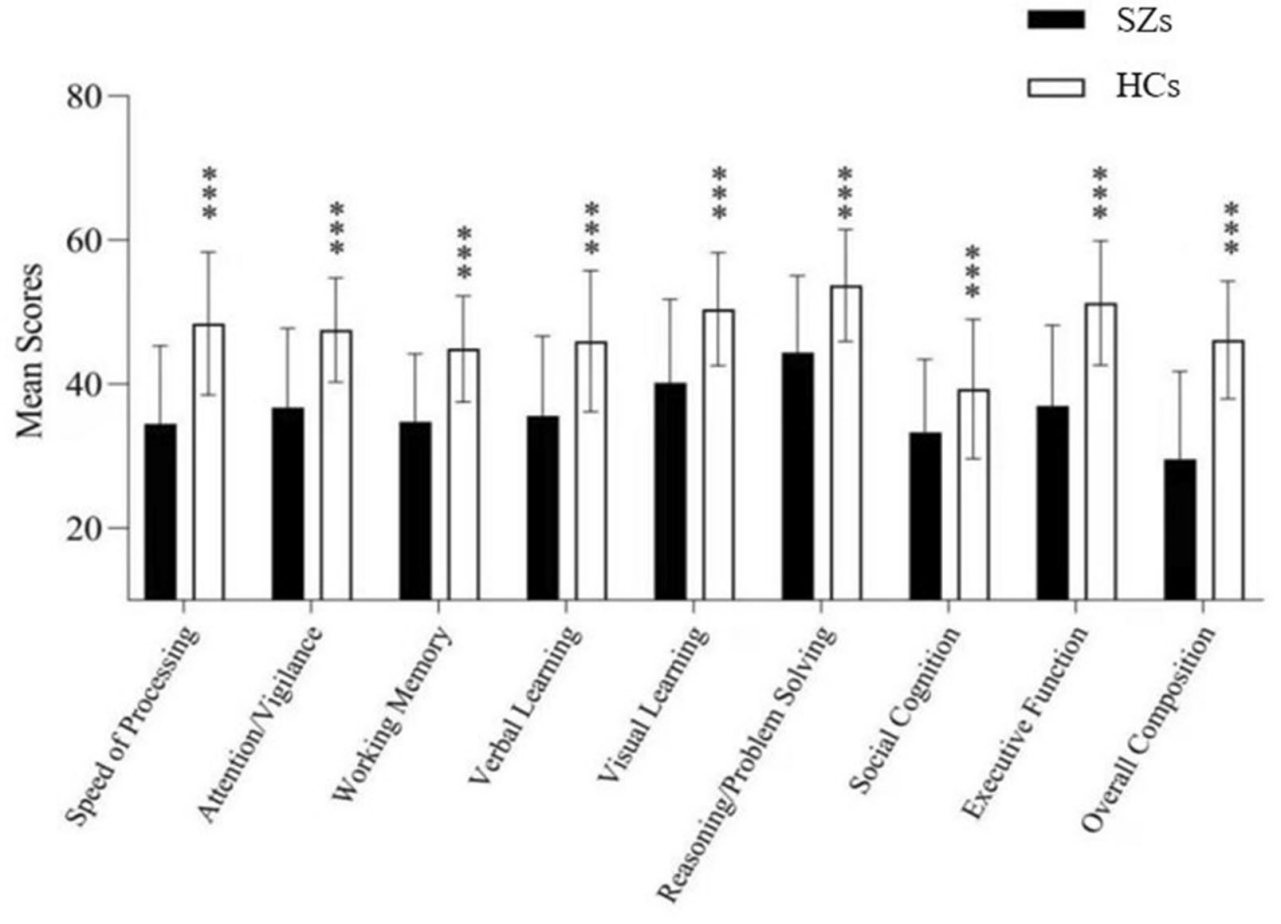
The difference in each cognitive domain of MCCB between the patients with SZ and HCs (*p* < 0.001). Seven cognitive domain scores in the patients with SZ and HCs. The patients with SZ exhibited significantly lower scores in all seven cognitive domains than HCs. ***p < 0.001 compared with healthy controls at the same cognitive domain

One hundred and seventeen patients with drug-naive first-episode SZ and 98 HCs were included. Table 1 presents the demographic and clinical data of all the patients. The SZ patient group exhibited a lower education level and a lower percentage of women than the HC group (*t'* = − 2.31, *p* = 0.22; χ^2^ = 0.32, *p* = 0.030, respectively). No significant difference was observed in the age between these two groups (*p* > 0.05). Both the total score and subscore in MCCB were lower in the SZ patient group than in the HC group (all *p* < 0.001) (Fig. 1).

**Table 1:** Sociodemographic and neonatal characteristics and results of the neuropsychological assessment of the SZs and HCs

	SZs (*n* = 117)	HCs (*n* = 98)	Statistic	
	Mean ± SD	Mean ± SD	*t*/*t'*/*X* ^2^	*p*
Age (years)	24.73 ± 7.01	26.53 ± 7.05	*t'* = − 0.47	0.062
Gender (male/female)	86/31	58/40	*X *^*2*^ = 0.32	0.030
Handedness (right/left)	115/0	98/0		
Years of education	13.18 ± 2.79	14.14 ± 2.31	*t'* = − 2.31	0.022
DUP (months)	15.77 ± 14.56	NA		
WAIS (IQ)	102.80 ± 11.69	116.67 ± 9.15	*t'*= − 8.17	0.000
Cognitive domains				
Speed of processing	33.52 ± 10.25	49.36 ± 9.74	*t'* =− 9.86	0.000
Attention/vigilance	36.12 ± 10.82	47.96 ± 7.36	*t'* = − 7.88	0.000
Working memory	34.12 ± 9.67	45.12 ± 7.12	*t'* = − 7.97	0.000
Verbal learning	35.16 ± 11.08	46.70 ± 9.77	*t'* = − 6.86	0.000
Visual learning	39.43 ± 11.86	50.73 ± 8.04	*t'*= − 6.87	0.000
Reasoning/problem solving	43.57 ± 10.69	54.45 ± 7.06	*t'* = − 7.39	0.000
Social cognition	32.69 ± 9.87	39.56 ± 9.16	*t'* = − 4.49	0.000
Executive function	36.30 ± 11.01	52.33 ± 8.50	*t'* = − 10.07	0.000
overall composite	28.55 ± 12.23	47.00 ± 7.53	*t'* = − 11.46	0.000
PANSS				
All totals	91.48 ± 7.88	NA		
Positive symptoms	16.16 ± 2.80	NA		
Negative symptoms	18.95 ± 3.20	NA		
General	46.13 ± 3.83	NA

### Cortical thickness comparisons between the groups

**Fig. 2:**
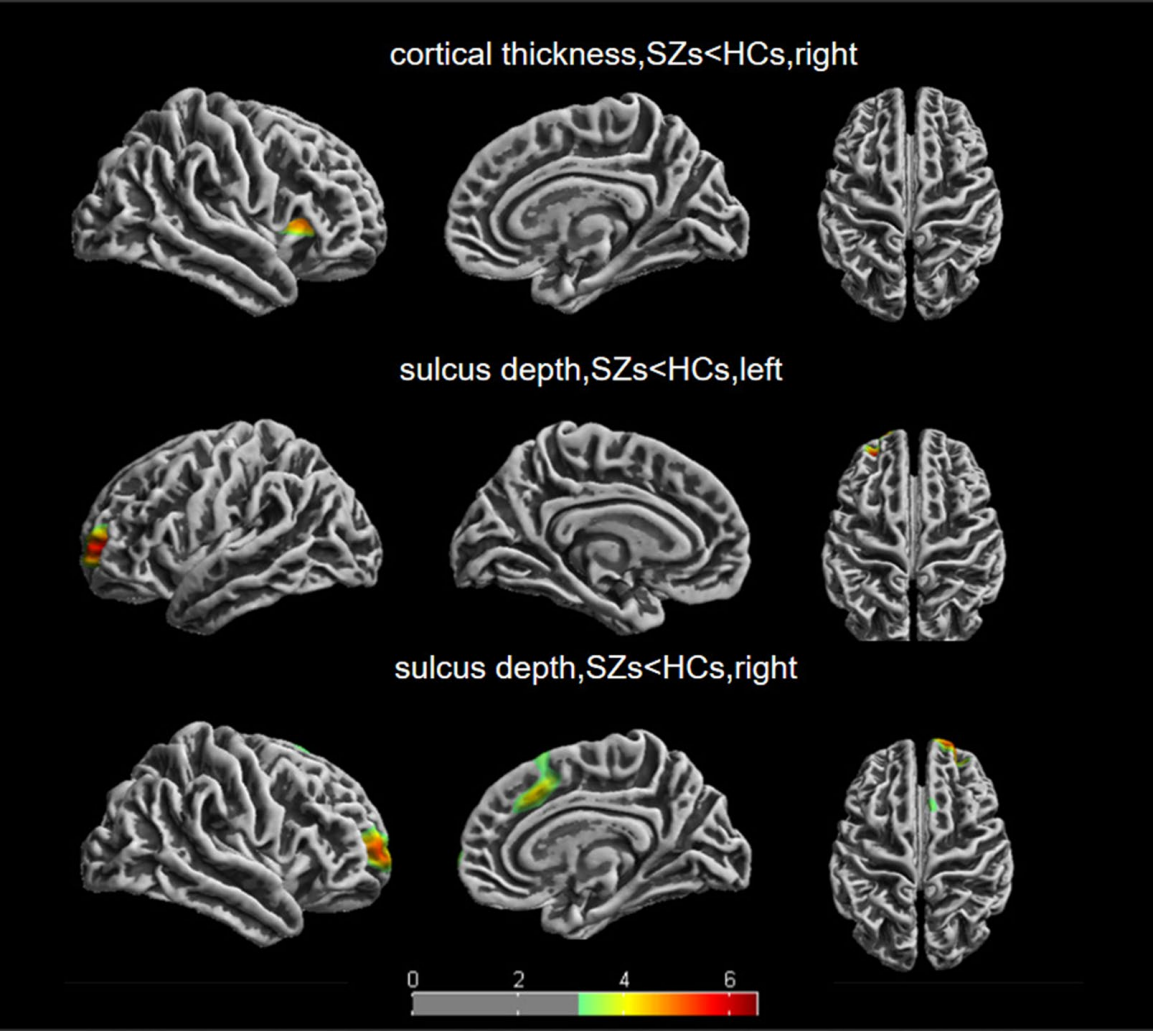
Differences in cortical thickness and sulcus depth between the patients with SZ and HCs. Results of between-group two-sample *t* tests; FWE correct, *p* < 0.05. The colour bars represent *t* values

Table 2 presents the cortical thickness difference between the patients with SZ and HCs evaluated using ANCOVA, with age, sex, and education years as covariates. Patients with SZ exhibited reduced thickness in the right insula and right pars triangularis, right pars opercularis, right lateral orbitofrontal, and right precentral cortices (all *p* < 0.05, FWE correction). Figure 2 is an excerpt from the original map of CAT12, illustrating the specific differential brain regions.

**Table 2:** Significant clusters showing group differences in cortical thickness (FWE correction, *p* < 0.05)

Cortical region (hemisphere)	Cluster size, vertices	*p* value	Coordinates of the max		
			*x*	*y*	*z*
33% insula (R)	1763	0.000	34	24	9
29% pars triangularis (R)					
28% parsoperculairs (R)					
9% later orbitofrontal (R)					
1% precentral (R)					

This chart is Desikan–Killiany DK40 Atlas (L: left, R: right)

### Sulcus depth comparisons between the groups

In Table 3, the patients with SZ exhibited lower sulcus depth in six regions, namely left rostral middle frontal, left rostral middle frontal, left superior frontal, right rostral middle frontal, right superior frontal, and right frontal pole, than HCs (all *p* < 0.05, FWE correction). Figure 2 is an excerpt from the original map of CAT12, illustrating the specific differential brain regions.

**Table 3:** Significant clusters showing group differences in sulcus depth (FWE correction, *p* < 0.05)

Cortical region (hemisphere)	Cluster size, vertices	*p* value	Coordinates of the Max		
			*x*	*y*	*z*
92% rostral middle frontal (L)	1569	0.000	− 24	52	5
8% superior frontal (L)			− 23	51	5
56% rostral middle frontal (R)	1470	0.000	25	61	7
58% superior frontal (R)			31	49	7
2% frontal pole (R)					
100% superior frontal (R)	1363	0.001	7	23	40

This chart is Desikan–Killiany DK40 Atlas (L: left, R: right)

### Correlation analysis

**Fig. 3:**
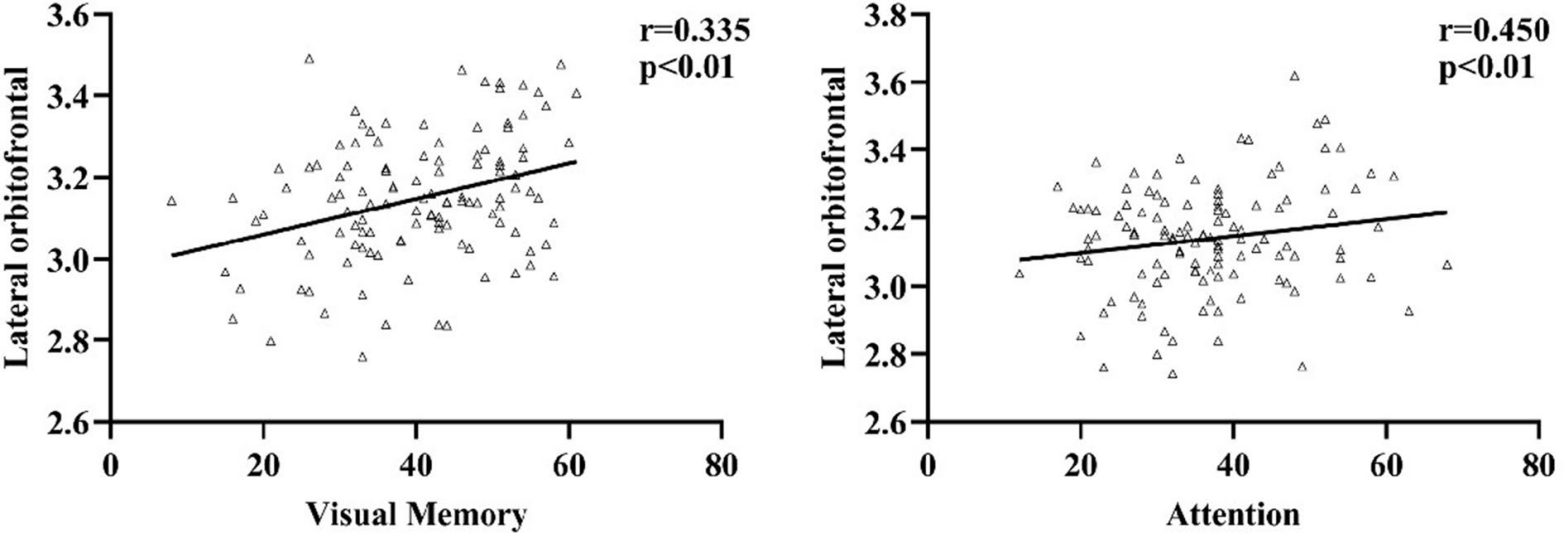
Pearson correlation analyses exhibited that the cortical thickness decreased in the lateral orbitofrontal gyrus of patients with SZ and demonstrated correlation with attention and visual learning

The abnormal brain region values in the patient group were extracted. Partial correlation analysis was used to determine the correlation among clinical data after correcting for age, sex, and education. The right lateral orbitofrontal cortical thickness was positively correlated with the attention and visual learning in patients with SZ (*r* = 0.450, *p* < 0.01, *r* = 0.335, *p* < 0.01, respectively) (Fig. 3). Additionally, no significant correlation was observed between sulcus depth and cognitive outcomes in the present study. No significant correlation was observed between cortical thickness or sulcus depth and the PANSS subscale score in the patients with SZ (*p* > 0.05). Additionally, correlation analyses revealed no obvious associations between the mean values of cortical surface and cognitive domain scores in the HCs.

## Discussion

The cognitive function, cortical thickness, and sulcus depth in different brain regions were compared between the patients with SZ and HCs. Additionally, the correlation of the altered cortical thickness and sulcus depth with cognitive deficits in patients with SZ was analysed. Compared with HCs, the patients with SZ exhibited extensive cognitive impairments, including those related to attention, visual learning, and working memory. Moreover, the patients with SZ exhibited lesser cortical thickness in five brain regions (namely the right insula, right pars triangularis, right pars opercularis, right lateral orbitofrontal, and right precentral) and lower sulcus depth in six brain regions (namely the left rostral middle frontal and left rostral middle frontal, left superior frontal, right rostral middle frontal, right superior frontal, and right frontal pole) than HCs. The cortical thickness in the right lateral orbitofrontal was positively correlated with cognition, particularly attention and visual learning, in the patients with SZ.

Numerous studies have reported several cognitive impairments in patients with schizophrenia [22], especially in chronic patients with a long disease course [23, 24]. However, the mechanism of cognitive damages in schizophrenia remains unknown [25]. Studies have exhibited that although the long-term antipsychotic use can help in improving mental symptoms and disease control, it may cause cognitive impairment in patients [26]. Therefore, antipsychotics may be a crucial factor for cognitive impairment. However, some studies have indicated significant improvements in individuals in the prodromal stage of schizophrenia without medication [7, 27]. Although the results of these studies are inconsistent, MCCB has often been considered as the current mainstream tool for identifying cognitive function damages in the early stage of schizophrenia [11, 28]. Additionally, the GEOPTE scale, in which the subjective experience of patients is used for evaluating cognitive functions, especially social functioning, exhibits superior psychometric behaviour in terms of both internal consistency and correlation with clinical global variables, mood, and degree of insight [29]. Additionally, the cognitive function, particularly the social function, has vital clinical significance, including in predicting disease development and evaluating patient recovery, for young patients with psychiatric disorders and other high-risk groups. Targeted specialised interventions can also improve the social function of early psychosis and improve the quality of life of patients [30, 31]. The medications and long disease course can be reduced, and the pathological mechanism can be uncovered by studying the characteristics and possible mechanisms of cognitive impairment in first-episode untreated patients, which can further promote drug research and development.

Compared with HCs, the patients with SZ exhibited increased reduction in the cortical thickness in several brain regions, including the right insula, right pars triangularis, right pars opercularis, right lateral orbitofrontal, and right precentral. A study exhibited that the thickness of the left insula and superior temporal gyrus in patients with SZ was significantly thinner than that in HCs [32]; however, the study did not assess the cognitive function. Additionally, other studies have exhibited a significant decrease in cortical thickness, mainly in the frontal and temporal lobes, in patients with schizophrenia compared with that in HCs [33]. Several studies have suggested that the structural changes in the frontal and the insula lobe may be linked to schizophrenia pathogenesis. These findings are concurrent with those of the present study. Animal studies have also exhibited that the use of antipsychotics can modulate the frontal lobe function, thereby improving psychotic symptoms [34]. This finding further suggests that antipsychotics may improve the clinical symptoms of patients with schizophrenia by regulating the structure or function of the frontal cortex. In the present study, variations in cortical thickness in the right lateral orbitofrontal were associated with cognitive impairment, including attention and visual learning, in patients with SZ. Another study exhibited that the loss of grey matter in bilateral superior temporal gyrus and left frontal lobe in patients with schizophrenia may be related to attention, which may reflect as disease-related grey matter damage in patients with SZ, thus disrupting the functional–structural relationship [35]. Some studies have exhibited a diagnostic interaction between verbal IQ and the thickness of right temporo-occipital junction and the left middle occipital gyrus in patients with schizophrenia [36]. Patients with schizophrenia also exhibit an association between working memory and right medial and superior temporal cortex thickness, suggesting that these patients may use a larger compensatory network of brain regions outside the frontal cortex for working memory [37]. Findings of some of the aforementioned studies are inconsistent with our results, which may be due to differences in the sample size, drug administration, or disease course. However, a consensus exists on the changes in the cerebral cortical structure in schizophrenia, and these changes may be associated with cognitive impairment.

Patients with SZ exhibited changes in sulcus depth, mainly in the left rostral middle frontal, left rostral middle frontal, left superior frontal, right rostral middle frontal, right superior frontal, and right frontal pole. Several studies focusing on the olfactory sulcus have reported that variations in the olfactory sulcus depth that existed before the onset of psychosis can predict the transition of psychosis. Additionally, these studies have indicated that the morphology of the olfactory sulcus changes during the disease course [38, 39]. These studies may not be comprehensive enough to study the neurodevelopmental mechanism of schizophrenia owing to the limitation of the studied brain regions and the sample inconsistency. A study indicated that the gestational disruption underlying schizophrenia is likely to predate, and the burden of cognitive damages may be associated specifically with the aberrant superior frontal development, which is apparent in late second trimester [40], suggesting that brain sulcus depth changes in the olfactory and superior frontal may be involved in the development of schizophrenia. Additionally, the frontal lobe plays a critical role in the integration and regulation of higher neural activities in humans [41]. Furthermore, a study exhibited that abnormal spontaneous activities in the right middle frontal gyrus indicate the severity and cognitive damages of disease [42]. The present study observed that the sulcus depth changes in patients with SZ were concentrated in the frontal lobe compared with those in HCs. Only a few studies have investigated the cerebral sulcus and cognitive impairment in schizophrenia, and the association between abnormal sulcus depth and cognitive impairment has not been reported. The present study exhibited that both cortical thickness and sulcus depth abnormalities in schizophrenia are concentrated in the prefrontal lobe. This finding is concurrent with those of other studies and indicates a possible correlation between cortical thickness and cortical complexity. Moreover, the present study excluded the effects of drugs and long disease duration, which might have increased the reliability of the study findings.

Additionally, previous studies have found that patients with schizophrenia demonstrate varying degrees of metabolic abnormalities, including those related to thyroid hormones, steroids, and glucose metabolism. Some studies have reported that patients with first-episode psychosis have reduced thyroid-stimulating hormone levels, although thyroid-stimulating hormone level was shown to be increased in patients with multiple-episode schizophrenia [43]. Some studies suggest that the sex hormone level in schizophrenia reflects the disease severity. Moreover, the serum progesterone level was reported to be negatively correlated with the PANSS total score and PNASS positive score [44]. Mildly elevated prolactin levels were associated with higher total PANSS scores in first-episode untreated women with schizophrenia [45]. Some scholars have speculated that the oxidation–antioxidant imbalance may be one of the influencing factors leading to mild cognitive impairment in schizophrenia [46]. The present study indicates that patients with schizophrenia exhibit changes in the endocrine and metabolic levels, which may affect the cognitive level of these patients. This suggests that we need to pay attention to the relationship between metabolic changes and brain structure and cognition in schizophrenia.

### Limitations

The present study has certain limitations. First, the effects of drugs and course of disease were not studied; hence, our cognitive evaluation was not detailed enough. Second, the cross-sectional design of the study prevented the prediction of neurobiological factors that may affect the SZ outcome. Moreover, some studies have exhibited that the cortical thickness decreases with age in healthy individuals, and therefore, attenuated group differences should have been considered in this research. Simultaneously, the inclusion of high-risk groups of schizophrenia and longitudinal analysis of their data would have been helpful in identifying the sensitive factors for schizophrenia. Finally, we chose the commonly used parameters in the MRI data processing, which may exhibit a few effects on the image.

## Conclusions

Local reductions in the cortical thickness and sulcus depth, particularly in the superior frontal, lateral orbitofrontal, and post central regions, were observed in patients with SZ. This finding provides evidence for structural abnormalities in SZ. Additionally, the association of cortical thickness with cognitive ability and mental health outcomes was observed. Therefore, this study suggests that the cortical structure may provide biomarkers for SZ.

## Data Availability

The data sets used and/or analysed during the present study are available from the corresponding author on reasonable request.

## Abbreviations

MRI: Magnetic resonance imaging
SZs: Drug-naive first-episode schizophrenia
HCs: Healthy controls
SBM: Surface-based morphometry
MCCB: Chinese version of the MATRICS Consensus Cognitive Battery
PANSS: The Positive and Negative Syndrome
FEW: Family-wise error
VBM: Voxel-based morphometry
DUP: Duration of untreated psychosis
WAIS: Wechsler Adult Intelligence Scale
IQ: Intelligence quotient
MCCB: The MATRICS Consensus Cognitive Battery
PANSS: Positive and Negative Syndrome Scale
NA: Not applicable
FWHM: Full width at half maximum
ANOVA: The analysis of variance
MECT: Modified electroconvulsive therapy
